# The value of fragmented QRS in predicting the prognosis of chronic total occlusion patients with myocardial infarction history undergoing percutaneous coronary intervention: A 24‐months follow‐up study

**DOI:** 10.1002/clc.23573

**Published:** 2021-02-16

**Authors:** Tiangui Yang, Xi Fu, Peng Fu, Jie Chen, Changlu Xu, Xiaoxia Liu, Tiesheng Niu

**Affiliations:** ^1^ Department of Cardiology Shengjing Hospital of China Medical University Shenyang Liaoning China

**Keywords:** chronic total occlusion, fragmented QRS, major adverse cardiovascular events, percutaneous coronary intervention

## Abstract

**Background:**

Fragmented QRS (fQRS) is a marker of local myocardial scar. This study aimed to analyze the relationship between fQRS and coronary collateral circulation (CCC) and evaluate the predictive value of fQRS for long‐term clinical outcomes among patients with chronic total occlusion (CTO) and prior myocardial infarction (MI) who underwent percutaneous coronary intervention (PCI).

**Methods:**

A total of 862 patients with a definite history of MI who had one CTO coronary artery and underwent PCI between 2013 and 2018 were continuously analyzed. Patients were divided into group A (no Q wave and fQRS, *n* = 206), group B (fQRS, *n* = 265), group C (Q wave, *n* = 391). All patients were followed up for 2 years.

**Results:**

The incidence rate of major adverse cardiovascular events (MACE) in group B was significantly lower than in group C (group B vs. C: 7.2% vs. 11.3%, *P* = 0.043). The percentage of good CCC was 94.2%, 88.3%, and 82.9% in group A, B, and C (*p* < .001), respectively. The improvement of cardiac function in group B and A were more significant than in group C. Multivariate Cox regression analysis showed fQRS was an independent protective factor of MACE after PCI within 2 years in CTO patients with prior MI (RR = 0.668, 95% CI [0.422–0.917], *p* = .001).

**Conclusion:**

fQRS is an independent protective factor of prognosis in patients with prior MI and one CTO vessel who underwent PCI, presenting with a higher rate of good CCC, less occurrence of MACE, and better heart function than in Q wave patients.

**Abbreviations**
6MWT: 6‐minute walking test
ACEI: angiotensin‐converting enzyme inhibitor
ACS: acute coronary syndrome
ARB: angiotensin receptor blocker
BMI: body mass index
CABG: coronary artery bypass grafting
CAD: coronary artery disease
CCC: coronary collateral circulation
CHF: congestive heart failure
CRT: cardiac resynchronization therapy
CTO: chronic total occlusion
fQRS: fragmented QRS
IVUS: intravascular ultrasound
LAD: left anterior descending
LCX: left circumflex
LVEDVI: left ventricular end diastolic volume index
LVEF: left ventricular ejection fraction
MA: malignant arrhythmia
MACE: major adverse cardiovascular events
MI: myocardial infarction
PCI: percutaneous coronary intervention
PTCRA: percutaneous transluminal coronary rotational atherectomy
RCA: right coronary artery
SCI: sudden cardiac death
ST: stent thrombosis

## 1 INTRODUCTION

Chronic total occlusion (CTO) of the coronary artery is a serious type of coronary artery disease (CAD) relatively difficult to treat.^1^, ^2^, ^3^ When the coronary artery is completely occluded, it leads to long term myocardial ischemia, eventually resulting in some necrotic or hibernating myocardium.^3^, ^4^, ^5^, ^6^ In a considerable number of patients with CAD, coronary angiography reveals at least one coronary artery with chronic total occlusion.^7^, ^8^, ^9^ Many studies have shown that the CTO of the coronary artery is associated with cardiac dysfunction.^10^, ^11^ Some studies have found that the cardiac function and quality of life can be improved to a certain extent after opening occluded vessels in CTO patients.^12^, ^13^, ^14^ Many patients with a history of myocardial infarction (MI) underwent at least one coronary artery with CTO and experienced cardiac dysfunction due to failure to timely open the occluded artery.^15^ Although the technology of opening CTO vessels gradually matured, and the success rate of opening CTO vessels has been significantly improved, some studies still believe that opening CTO vessels does not bring obvious benefits to CTO patients.^16^, ^17^, ^18^ At present, some studies advocate that although the patients' heart function can be improved after opening the CTO coronary artery, the incidence of adverse cardiovascular events (MACE) has not been reduced.^17^, ^18^, ^19^, ^20^ Whether opening CTO vessels is beneficial to patients varies from person to person, and different types of patients experience benefits of different extent. Many factors can promote cardiac function change and the occurrence of MACE in CTO patients after the percutaneous coronary intervention (PCI).^19^, ^20^, ^21^

The evidence of prior transmural MI are the pathological Q‐waves on standard 12‐lead electrocardiographic (ECG).^22^ Q‐waves on ECG can independently predict the occurrence of MACE. In their study, Ömer Kozan et al ^23^, ^24^ showed that Q wave amplitude/precordial total R wave amplitude ratio (Q/R) in admission ECG could predict in‐hospital outcomes, especially the no‐reflow in patients with first acute anterior MI treated with primary PCI. There is no established ECG sign for a prior MI in the absence of pathological Q‐wave on ECG, while some patients with a history of myocardial infarction may even have normal ECG.^22^, ^25^ Recently, it has been shown that the fragmented QRS (fQRS) on ECG signifies regional myocardial scar in patients with non‐Q‐wave MI.^22^, ^25^ FQRS can represent the formation of scar in local myocardial tissue and reflect the presence of island‐like viable myocardium in the area of myocardial scar.^25^, ^26^ Studies found that fQRS is closely associated with acute coronary syndrome, arrhythmias such as ventricular arrhythmia, early cardiac dysfunction, the reactivity of cardiac resynchronization therapy (CRT), and sudden cardiac death (SCD).^26^, ^27^, ^28^ fQRS was also reported as a significant independent predictor for cardiac events and cardiac mortality in NSTEMI patients.^29^ Recent studies by Kurtul et al^30^, ^31^ found that fQRS was an independent predictor of postprocedural contrast‐induced nephropathy and in‐hospital mortality in STEMI patients. Moreover, fQRS could predict the development of postoperative atrial fibrillation (PoAF) in patients undergoing coronary artery bypass grafting (CABG) surgery. Nonetheless, it still remains unclear which approach is more suitable to open CTO vessels for prior MI patients with fQRS, Q wave, no Q wave, and fQRS on ECG. Although some studies have shown that fQRS on ECG may be related to poorly developed collateral circulation in patients without prior MI, few studies have focused on patients with a definite history of MI.^22^, ^27^, ^28^ Unfortunately, the predictive value of fQRS for the change of cardiac function and MACE after PCI in CTO patients with prior MI is not clear.^32^

The purpose of this study was to investigate the relationship between fQRS and coronary collateral circulation (CCC), and also to evaluate the predictive value of fQRS in the change of cardiac function and the incidence of MACE after PCI in CTO patients with prior MI.

## 2 METHODS

### 2.1 Participants

A total of 862 patients with a definite history of MI who were hospitalized in Shengjing Hospital of China Medical University between January 2013 and June 2018 were analyzed in this prospective study. Written informed consent was obtained from all enrolled MI inpatients. The study was approved by the Ethics Committee of Shengjing Hospital and was performed in accordance with the Declaration of Helsinki.

First, 3750 patients with a definite history of MI were screened, including 1045 patients with one confirmed CTO coronary artery who underwent PCI. Patients with two or more CTO coronary arteries were excluded. We collected the demographic characteristics, previous medical history and family history, laboratory examination, drug treatment, interventional imaging data, ECG characteristics, complications during hospitalization of all patients. Full follow‐up notification was made to all patients, including telephone follow‐up, outpatient follow‐up, and re‐hospitalization reexamination. We observed the occurrence of MACE and the change in cardiac function over the period of 2 years after PCI for all patients. Finally, 862 patients (mean age 64.5 ± 2.8, 53.2% male) were included and analyzed. Patients were divided into three groups: Group A (no Q wave and fQRS, *n* = 206), Group B (fQRS, *n* = 265), Group C (Q wave, *n* = 391) according to the presence of fQRS or Q wave on ECG.

### 2.2 Exclusion criteria

(1) Acute myocardial infarction within 1 month; (2) patients without a history of myocardial infarction; (3) there were two or more CTO coronary arteries; (4) previous history of coronary artery bypass grafting (CABG) or PCI; (5) history of preexcitation syndrome or pacemaker implantation; (6) left bundle branch block, complete or incomplete right bundle Branch block; (7) rheumatic heart disease, dilated cardiomyopathy, hypertrophic cardiomyopathy, acute myocarditis, congenital heart disease; (8) cardiogenic shock, malignant arrhythmia, serious blood system disease, malignant tumor, serious liver and kidney disease; (9) history of stroke in the last 6 months, and history of serious trauma, serious infection, and operation in the last 3 months.

### 2.3 Coronary angiography and coronary collateral scoring

The coronary intervention was performed as follows: all patients were given aspirin, clopidogrel or ticagrelor, and statins before PCI. After coronary angiography, all patients underwent balloon dilatation and stent placement. Criteria of recanalization: the diameter of the target vessel was <50% after balloon dilatation, or the diameter of the target vessel was <20% after coronary stenting. The forward blood flow reached TIMI 3, and there were no serious complications. Routine treatment of antiplatelet, lipid‐lowering was continued after PCI. Coronary collateral circulation was graded according to Rentrop's classification: Grade 0‐no visible collaterals, Grade 1‐filling of side branch via collateral vessels without visible epicardial coronary artery, Grade 2‐incomplete filling of the epicardial coronary artery, and grade 3‐complete filling of the epicardial coronary artery. Grades 2 and 3 are considered as good CCC.^17^, ^18^, ^20^ Evaluation of angiographies, and collateral scoring was performed by two experienced cardiologists not involved in the present study.

### 2.4 Definition

#### 2.4.1 Definition of fQRS

The definition of fQRS^27^, ^28^, ^32^, ^33^ was first proposed by Mithilesh and his team in 2006.^33^ fQRS refers to the three‐phase or polyphase QRS waves that appear or exist in the electrocardiogram of patients with coronary heart disease and myocardial infarction. They have the following basic characteristics (Figure S1): (1) QRS waves are three‐phase or polyphase waves, some of which are characteristic of RSR type, in which polyphase waves are often formed by multiple setbacks or notches of R waves or S waves; (2) with or without Q waves, they can form QR or QR type; (3) most of QRS wave time limit <120 ms; (4) no complete or incomplete bundle branch block and intraventricular conduction block; (5) three‐phase or multi‐phase QRS fragmentation often occurs in two or more leads corresponding to the coronary blood supply area; (6) different leads of the same ECG in the same patient may show different forms of QRS fragmentation. All ECGs were analyzed by two independent cardiologists (Figure S1).

#### 2.4.2 MACE

The MACE^34^, ^35^ after PCI includes: all‐cause death (including cardiac death), all myocardial infarction, any revascularization, definite/probable stent thrombosis (ST), malignant arrhythmia (MA), congestive heart failure (CHF), and ischemic stroke.

#### 2.4.3 6‐minute walking test

A 6‐minute walking test (6MWT)^36^, ^37^ with the specific evaluation method was as follows: patients were told to complete the farthest walking distance in 6 min, monitor vital signs before and after the exercise test, and stop the test immediately in case of obvious symptoms. The walking distance was measured and recorded at the end of the test at 6 min. The experiment was immediately stopped in case of the following conditions: (1) foot pain; (2) sweating; (3) faltering; (4) spasm of lower limbs; (5) intolerable dyspnea; (6) pale face; (7) other symptoms preventing the patient from completing the experiment.

### 2.5 Statistical methods

SPSS 20.0 software was used for statistical analysis. Continuous variables conforming to normal distribution were expressed as mean ± SD, and *t* tests were used for comparison between groups. Continuous variables were compared using a one‐way analysis of variance followed by post hoc analysis using the Bonferroni correction test for multiple inter‐group comparisons. Variables with were non‐normal distribution indicated using median (Interquartile Range, IQR) and were compared between groups by a nonparametric test using Kruskal‐Wallis H test. Categorical variables were presented as counts and percentages (%), categorical variables were compared using the *χ* ^2^ test and Fisher exact probability test was used to analyze their inter‐group comparisons. Analysis of variance was used to analyze the improvement of cardiac function after PCI among three groups. Unadjusted cumulative event rates were estimated using the Kaplan–Meier method and compared among groups using the log‐rank test. In order to estimate the possible associations between fQRS and MACE, multivariate Cox regression analyses were performed by adjusting the variables with statistically significant (*p* < .05) comparisons and combined with clinical consideration simultaneously. Adjusted variables included Q wave, no Q wave, and fQRS, good CCC, smoking, hypertension, diabetes, family history of myocardial infarction, male, age > 65y, mean stent diameter, mean stent length, left ventricular ejection fraction (LVEF), low‐density lipoprotein (LDL). *p* < .05 suggested statistically significant difference.

## 3 RESULTS

### 3.1 Comparison of basic data

The comparison of basic data among the three groups is shown in Table 1. The proportion of smoking and diabetes in Group B was higher than that in group A and C (Group A vs. B vs. C: Smoking, 54.4% vs. 58.1% vs. 47.3%; Diabetes, 34.5% vs. 41.1% vs. 35.0%, *p* < .05). In addition, group C had more males, and the proportion of family history of MI in group C was higher than in group A and B (group A vs. B vs. C: Male, 49.5% vs. 49.4% vs. 57.8%; Family history of MI, 4.4% vs. 6.8% vs. 9.5%, *P* < .05). There were more CTO of RCA in group B (RCA vs. LAD vs. LCX: 47.5% vs. 36.6% vs. 15.9%, *p* < .001), while there was no difference between group A and C (Table 1).

**TABLE 1:** Comparison of general baseline data among three groups (^−^x ± s; median [IQR]; n, %)

Characteristic	Group A (no Q wave and fQRS, *n* = 206)	Group B (fQRS, *n* = 265)	Group C (Q wave, *n* = 391)	*p*‐value (All‐groups)
Basic data				
Age > 65 Y (*n*, %)	58 (28.2%)	81 (30.6%)	104 (26.6%)	.082
Male (*n*, %)	102 (49.5%)	131 (49.4%)	226 (57.8%)	.003
The course of MI (years)	6.4 (1.0)	6.5 (1.1)	6.4 (1.2)	.089
Smoking (*n*, %)	112 (54.4%)	154 (58.1%)	185 (47.3%)	.002
Hypertension (*n*, %)	98 (47.6%)	132 (49.8%)	180 (46.0%)	.374
The course of hypertension (years)	6.2 (1.0)	6.3 (1.1)	6.3 (1.2)	.495
Diabetes (*n*, %)	71 (34.5%)	109 (41.1%)	137 (35.0%)	.049
Course of diabetes (years)	6.8 (1.2)	6.9 (1.3)	6.7 (1.3)	.078
Cerebrovascular disease (*n*, %)	11 (5.3%)	18 (6.9%)	38 (9.7%)	.094
BMI (kg / m^2^)	25.4 ± 4.7	25.9 ± 4.8	25.7 ± 5.1	.277
Family history of MI (*n*, %)	9 (4.4%)	18 (6.8%)	37 (9.5%)	.043
Medication (*n*, %)				
Aspirin	206 (100%)	265 (100%)	391 (100%)	1
Clopidogrel	113 (54.9%)	150 (56.6%)	214 (54.7%)	.552
Ticagrelor	93 (45.1%)	115 (43.4%)	177 (42.3%)	.578
Statins	206 (100%)	265 (100%)	391 (100%)	1
Beta‐blocker	142 (68.9%)	196 (73.9%)	290 (74.2%)	.537
ACEIs/ARBs	129 (62.6%)	162 (61.1%)	260 (66.5%)	.324
Biochemical index				
CHOL (mmol/L)	4.7 ± 3.1	4.7 ± 2.9	4.9 ± 2.8	.342
TGs (mmol/L)	3.0 (0.9)	3.2 (1.0)	3.0 (1.1)	.515
LDL (mmol/L)	3.4 ± 1.7	3.3 ± 1.8	3.5 ± 1.8	.501
HbA1c (%)	7.3 ± 3.7	7.4 ± 3.5	7.2 ± 3.8	.281
FPG (mmol/L)	7.5 ± 4.7	7.6 ± 4.2	7.5 ± 4.5	.312
Cr (umol / L)	81.1 ± 22.9	84.1 ± 23.3	76.4 ± 25.1	.055
Distribution of CTO lesions (*n*, %)				<.001
LAD	64 (31.1%)	97 (36.6%)	123 (31.5%)	—
LCX	62 (30.1%)	42 (15.9%)	120 (30.7%)	—
RA	80 (38.8%)	126 (47.5%)	148 (37.8%)	—
***Rentrop Grade of CCC (n, %)***				<.001
Poor				
Grade 0 CCC	—	—	—	—
Grade 1 CCC	12 (5.8%)	31 (11.7%)	67 (17.1%)	—
Good				
Grade 2 CCC	16 (7.8%)	30 (11.3%)	72 (18.4%)	—
Grade 3 CCC	178 (86.4%)	204 (77.0%)	252 (64.5%)	—
NO. of stents per patient	3.0 (0.4)	3.0 (0.5)	3.0 (0.4)	.512
Mean stent diameter (mm)	3.4 (0.9)	3.5 (0.9)	3.4 (0.8)	.118
Mean stent length (mm)	52.7 ± 20.5	53.1 ± 21.8	54.1 ± 20.3	.411
Total procedure time (min)	114.1 ± 45.3	116.1 ± 51.3	114.8 ± 55.6	.159
Site of access (*n*, %)				.312
Femoral	174 (84.5%)	219 (82.7%)	316 (80.8%)	—
Radial	9 (4.4%)	12 (4.5%)	19 (4.9%)	—
Femoral and radial	23 (11.1%)	34 (12.8%)	56 (14.3%)	—
Successful technique (*n*, %)				.154
Forward wire Technique	141 (68.4%)	174 (65.7%)	261 (66.7%)	—
Reverse wire Technique	65 (31.6%)	91 (34.3%)	130 (33.3%)	—
IVUS used (*n*, %)	64 (31.1%)	85 (32.1%)	132 (33.8%)	.612
PTCRA used (*n*, %)	14 (6.8%)	17 (6.4%)	21 (5.4%)	.218

Abbreviations: ACEI, angiotensin‐converting enzyme inhibitor; ARB, angiotensin receptor blocker; BMI, body mass index; CCC, coronary collateral circulation; CHOL, total cholesterol; Cr, creatinine; CTO, chronic total occlusion; FPG, fasting plasma glucose; HbA1c, glycosylated hemoglobin A1c; IVUS, intravascular ultrasound; LAD, left anterior descending; LCX, left circumflex; LDL, low‐density lipoprotein; MI, myocardial infarction; PTCRA, percutaneous transluminal coronary rotational atherectomy; RCA, right coronary artery; TG, triglyceride.

### 3.2 Comparison of CCC grades

There were significant differences in the grade of CCC among the three groups (Group A vs. B vs. C: Grade 1, 5.8% vs. 11.7% vs. 17.1%; grade 2, 7.8% vs. 11.3% vs. 18.4%; grade 3, 86.4% vs. 77.0% vs. 64.5%. *p* < .001, Table 1). The proportion of good CCC (Grades 2 and 3) in group B was higher compared to group C, but lower than in group A (Group A vs. B vs. C: good CCC, 94.2% vs. 88.3% vs. 82.9%. *p* < .001, Table 1).

### 3.3 Change of cardiac function after PCI

In order to compare the changes in cardiac function within 2 years after PCI in the three groups, we compared the changes in 6MWT distance, brain natriuretic peptide (BNP), LVEF, and left ventricular end‐diastolic volume index (LVEDVI) at three different time points (Table 2). The cardiac function in all three groups improved within 2 years after PCI compared with before PCI. At 12 and 24 months of follow‐up time, the improvement of 6MWT and BNP in group B was similar to that in group A, which was better than that in group C (*p* < .001, Table 2). Although the improvement of LVEF and LVEDVI in group B was similar to that in group C at 12‐months follow‐up, which was worse than in group A (*p* = .002, Table 2), the improvement of LVEF and LVEDVI in group B was similar to that in group A at 24‐months follow‐up time, which was significantly better than that in group C (*p* = .001, Table 2).

**TABLE 2:** Change of cardiac function after PCI (x ± s)

Characteristic	Group A (no Q wave and fQRS, *n* = 206)	Group B (fQRS, *n* = 265)	Group C (Q wave, *n* = 391)	*p*‐value (A vs. B)	*p*‐value (A vs. C)	*P*‐value (B vs. C)	*p*‐value (All‐groups)
Before PCI							
6MWT (m)	379.6 ± 136.7	372.3 ± 134.9	367.4 ± 141.1	.315	.181	.214	.118
BNP (ng/ml)	315.3 ± 125.6	324.6 ± 134.6	345.6 ± 129.9	.129	.082	.064	.071
LVEF (%)	47.8 ± 7.9	47.1 ± 8.6	46.1 ± 9.0	.512	.296	.421	.112
LVEDVI (ml/m^2^)	80.1 ± 9.8	79.1 ± 10.2	81.1 ± 9.5	.452	.318	.311	.213
12‐months follow up							
6MWT (m)	451.6 ± 178.1	452.9 ± 186.1	415.3 ± 174.1	.418	.012	.011	<.001
BNP (ng/ml)	211.9 ± 109.4	226.6 ± 115.9	300.1 ± 119.4	.311	.009	.012	<.001
LVEF (%)	57.9 ± 8.7	53.6 ± 8.9	52.1 ± 9.4	.018	.011	.229	.001
LVEDVI (ml/m^2^)	74.4 ± 10.6	78.1 ± 10.1	78.6 ± 9.5	.021	.017	.312	.002
24‐months follow up							
6MWT (m)	495.3 ± 156.1	501.1 ± 166.9	462.6 ± 163.9	.412	.046	.031	<.001
BNP (ng/ml)	181.6 ± 102.7	189.2 ± 114.4	254.7 ± 127.1	.487	.008	.001	<.001
LVEF (%)	60.1 ± 8.5	59.1 ± 9.2	56.3 ± 7.9	.399	.016	.021	.002
LVEDVI (ml/m^2^)	70.6 ± 8.9	71.1 ± 9.5	75.1 ± 10.2	.316	.013	.008	.001

Abbreviations: 6MWT,6‐minute walking test; BNP, brain natriuretic peptide; LVEF, left ventricular ejection fraction; LVEDVI, left ventricular end‐diastolic volume index; PCI, percutaneous coronary intervention.

We listed the coronary angiography images before and after PCI of two patients who had fQRS on ECG, as well as the improvement in cardiac function indexes after PCI (24‐months follow‐up) (Figure S2). These two patients had typical fQRS on ECG (Figure S2A1, B1, red arrow). The angiogram indicates the corresponding CTO coronary before PCI (Figure S2A2, CTO of RCA; B2, CTO of LAD, blue arrow), the Rentrop grade of CCC in both cases were grade 3 (Figure S2A3, B3, blue arrow), and the blood flow was TIMI 3 after PCI (Figure S2A4, B4, blue arrow). The cardiac function in both cases significantly improved after PCI (Figure S2A5, B5).

### 3.4 Relationship between fQRS and MACE

**FIGURE 1:**
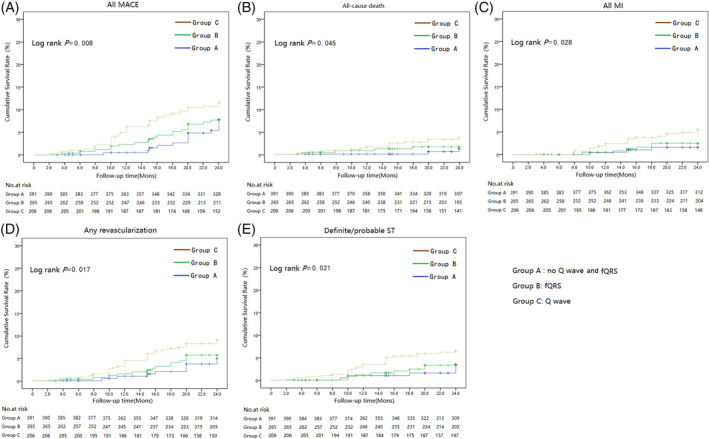
The Kaplan Meier curves for clinical outcomes. The cumulative incidence rate of all MACEs, all‐cause death, all MI, any revascularization, and definite/probable ST in group B was similar to that in group A; both of them were significantly lower than that in group C (All MACEs: Log‐rank *P* = 0.008, (A) All‐cause death: Log‐rank *p* = .045, (B) All MI: Log‐rank *p* = .028, (C) Any revascularization: Log‐rank *p* = .017, (D) Definite/probable ST: Log‐rank *p* = .021, (E) MACE: major adverse cardiovascular events; MI, myocardial infarction; ST, stent thrombosis

There were 77 patients (8.9%) with all MACEs over 2 years after PCI. The incidence rate of all MACEs in group B was higher than in group A, but there was no statistical difference (*p* = .390). Both A and B group had significantly lower incidence rates than group C (group A vs. B vs. C: 6.8% vs. 7.2% vs. 11.3%, *p* = .045). The incidence rate of all‐cause death, all MI, any revascularization, and define/probable ST were different between the three groups (*p* < .05), but there was no significant difference in the incidence rate between cardiac death, MA, CHF, and ischemic stroke (Table 3). The Kaplan Meier analysis also showed the same conclusion; the cumulative incidence rate of all MACEs, all‐cause death, all MI, any revascularization, and definite/probable ST in group B was similar to that in group A, both of which were significantly lower than that in group C (all MACEs: Log‐rank *p* = .008, A; all‐cause death: Log‐rank *p* = .045, B; all MI: Log‐rank *p* = .028, C; any revascularization: Log‐rank *p* = .017, D; definite/probable ST: Log‐rank *p* = .021, E. Figure 1).

**TABLE 3:** Long‐term clinical outcomes of patients in three groups up to 2 years (*n*, %)

Characteristic	Group A (no Q wave and fQRS, *n* = 206)	Group B (fQRS, *n* = 265)	Group C (Q wave, *n* = 391)	*p* value (A vs. B)	*p* value (A vs. C)	*p* value (B vs. C)	*p* value (All‐groups)
All MACEs (*n*, %)	14 (6.8%)	19 (7.2%)	44 (11.3%)	.390	.039	.043	.045
All‐cause death	2 (1.0%)	4 (1.5%)	13 (3.3%)	.259	.040	.049	.046
Cardiac death	2 (1.0%)	3 (1.1%)	10 (2.6%)	.454	.050	.054	.052
All MI	4 (1.9%)	6 (2.3%)	20 (5.1%)	.124	.026	.041	.031
Revascularization	9 (4.4%)	14 (5.3%)	34 (8.7%)	.175	.028	.039	.030
Definite/probable ST	6 (2.9%)	8 (3.0%)	24 (6.1%)	.526	.037	.044	.042
MA	1 (0.5%)	4 (1.5%)	12 (3.1%)	.119	.056	.127	.079
CHF	2 (1.0%)	3 (1.1%)	10 (2.6%)	.543	.212	.301	.245
Ischemic stroke	1 (0.5%)	2 (0.8%)	0	.398	.229	.312	.254

Abbreviations: CHF, congestive heart failure; MACE, major adverse cardiovascular events; MI, myocardial infarction; MA, malignant arrhythmia; ST, stent thrombosis.

### 3.5 Multivariate Cox regression analysis

**FIGURE 2:**
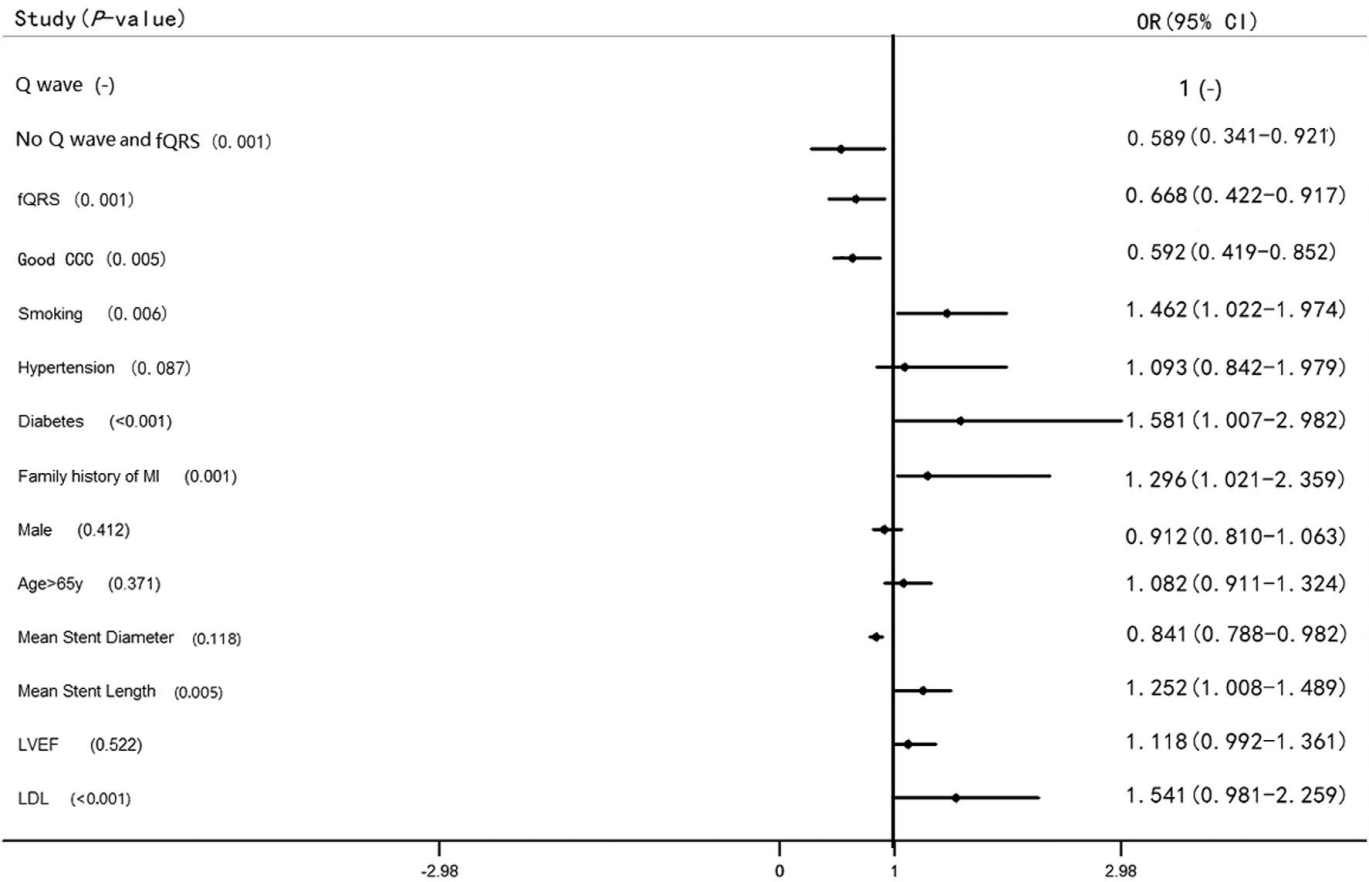
Multivariate Cox regression analysis. When adjusted for Q wave (group C), no Q wave and fQRS (group A), good CCC, smoking, hypertension, diabetes, family history of MI, male, age > 65y, mean stent diameter, mean stent length, LVEF, and LDL, fQRS is an independent protective factor for MACE within 2 years after PCI (RR = 0.668, 95% *CI* [0.422–0.917], *p* = .001). CCC, coronary collateral circulation; LDL: low‐density lipoprotein; LVEF: left ventricular ejection fraction; MACE, major adverse cardiovascular events; MI, myocardial infarction; PCI, percutaneous coronary intervention

To evaluate the predictive effect of fQRS on MACE after PCI, we included the factors that have been proven to influence MACE after PCI, as well as those that resulted statistically significant in the univariate Cox regression analysis into the multivariate Cox regression analyses model. We adjusted for Q wave (group C), no Q wave and fQRS (group A), good CCC, smoking, hypertension, diabetes, family history of MI, male, age > 65y, mean stent diameter, mean stent length, LVEF, LDL. fQRS resulted as an independent protective factor for MACE over 2 years after PCI in CTO patients with prior MI (RR = 0.668, 95% CI [0.422–0.917], *p* = .001, Figure 2).

## 4 DISCUSSION

The main findings of this study are as follows: the incidence rate of MACE in fQRS patients was significantly lower than in patients with Q wave (Group B vs. C: 7.2% vs. 11.3%, *p* = .043); multivariate Cox regression analysis showed that fQRS was an independent protective factor of MACE after PCI over 2 years in CTO patients with prior MI (RR = 0.668, 95% CI [0.422–0.917], *p* = .001); the proportion of good CCC and the improvement of cardiac function after PCI in fQRS patients were similar to those in no Q wave and fQRS patients, which were better than those in Q wave patients.

The occurrence of fQRS can indicate myocardial infarction or the presence of ischemic myocardium. According to previous studies, underlying mechanisms are mainly explained as follows^26^, ^27^, ^28^, ^32^, ^38^: block in the infarct area, block around infarct area, multifocal infarction, the theory of local myocardial scar, and change of intercellular impedance. The pathophysiological mechanism is as follows: (1) the conduction of the remaining viable myocardium in the infarcted area is slow due to ischemia, and the change of regional ventricular myoelectric activity affects the depolarization vector of the whole ventricle, thus making the ventricular electrical activity out of synchronized.^27^, ^38^ (2) regardless of endocardial mapping or nuclide angiocardiography load test, fragmentation potential is found in a large area around the myocardial scar. The myocardial fibrosis scar can interrupt the continuity of myocardial depolarization, thus changing the process and direction of ventricular depolarization, leading to the formation of fQRS. ^39^ (3) some studies suggest that fQRS is caused by the uneven activation of ventricular muscle due to myocardial scar and myocardial ischemia. There is island‐like viable myocardium in the area of the myocardial scar, and the surviving island myocardium is in a state of ischemia. As a result, the depolarization is delayed and slowly conducted, forming notch or setback of S wave that results in irregular fQRS. ^40^, ^41^ Over recent years, increasing attention has been paid to the concept of fQRS, which is related to various new situations and changes caused by the era of thrombolysis and coronary intervention. It has also been closely associated with the in‐depth study of fragmented QRS.^15^, ^16^, ^41^, ^42^ fQRS has important clinical significance as it can improve the diagnosis rate of prior MI and can be used as an early warning of high‐risk patients with MI.^30^, ^31^, ^42^

Our result revealed that the improvement of cardiac function in non‐Q wave groups (group A and B) was more significant than in Q wave group after PCI over 2 years in CTO patients with a history of MI. Although the proportion of good CCC (Grades 2 and 3) in fQRS patients was lower than in the patients without fQRS or Q wave, it was higher than in Q wave patients. This may be because, when the coronary artery is in progressive stenosis or even complete occlusion, the mechanism of myocardial ischemia protection promotes the formation of CCC,^43^ mainly through the mechanism of myocardial ischemia/reperfusion injury, the mechanism of myocardial cell protection, the mechanism of myocardial ischemic pretreatment and the mechanism of increasing the secretion of various growth factors.^26^, ^27^, ^40^, ^44^ FQRS complex has been explained by inhomogeneous activation of the myocardium due to myocardial scar or ischemia.^28^, ^32^, ^45^ Infarct size is inversely related to collateral circulation and directly related to the occlusion time. There may be no infracted myocardium within the occluded artery territory in about half of patients with chronic total occlusion.^40^, ^41^, ^45^ These patients may remain completely asymptomatic. After opening the CTO vessels in patients with fQRS on ECG, the ischemic and dormant myocardium are supplied with blood again. This part of the myocardium will recover its function, which can increase the myocardial blood supply and oxygen supply, thus improving the cardiac function and the quality of life of these patients.^44^, ^45^, ^46^ Also, with the extension of follow‐up time, the improvement in cardiac function may further increase, which shows that the blood supply of those ischemic myocardium becomes more and more sufficient with time.

This study also revealed that the incidence rate of MACE after PCI over 2 years in patients with fQRS on ECG was similar to that in patients without fQRS and Q wave, which was significantly lower than in patients with Q wave on ECG. This is because fQRS indicates the presence of scar tissue, especially non‐transmural scar, which contains temporarily disabled viable myocardium muscle (including hibernating myocardium and stunning heart). It has been found that the size, location, and transmural degree of scarring are significantly related to left ventricular volume and left ventricular ejection fraction.^27^, ^28^, ^32^, ^40^ Myocardial scar can be divided into the transmural scar and non‐transmural scar.^26^, ^27^, ^40^, ^45^ Unlike transmural scar, which almost has no residual myocardial tissue and produces Q wave or QS wave, there are a large number of viable myocardium in non‐transmural scar tissue.^47^ Viable myocardium includes hibernating myocardium and (or) stunning myocardium, which can be reperfused and can improve the movement of surviving myocardium and promote the partial or even complete recovery of cardiac function after PCI revascularization.^41^, ^42^, ^48^ It has been reported that the successful opening of the completely occluded coronary artery can not only relieve the patients' angina symptoms but also stabilize the electrical activity of the myocardium, improve the left ventricular function, and further enhance the patients' tolerance to various adverse events in the future.^46^, ^47^, ^48^, ^49^, ^50^ Therefore, it is very important to open the CTO vessels as soon as possible in patients with fQRS on ECG.

## 5 CONCLUSION

This study indicated that fQRS was an independent protective factor of prognosis in patients with prior MI and one CTO vessel post PCI, presenting with a higher rate of good CCC, less occurrence of MACE, and better heart function compared to Q wave patients. fQRS could be used as a cheap and convenient indicator for selecting suitable CTO patients for PCI.

## 6 LIMITATIONS

Although fQRS can reflect the existence of viable myocardium in the myocardial scar to a certain extent,^26^, ^27^, ^28^ it needs to be detected by MRI and SPECT examination, which can more directly and fully reflect the situation of viable myocardium.^32^, ^33^ In this study, SPECT or MRI could not be done for all patients; thus, we could only observe the relationship between the proportion of fQRS on ECG, cardiac function, clinical biochemical indicators, and MACEs. Further studies with larger numbers of subjects using MRI are warranted to further clarify this issue. Secondly, the sample size in our study was small due to the consideration of individually selected cases only. Our results need to be confirmed by large‐scale, prospective, and long‐term follow‐up studies. We can formulate more intensive drug treatment strategies for patients with fQRS on ECG, even implement ICD and CRT, and then further observe whether these treatment strategies are effective for reducing the incidence of MACE after PCI in CTO patients with prior MI.

## CONFLICT OF INTEREST

None of the authors have any conflict of interest to report. Neither the entire paper nor any part of its content has been published or has been accepted elsewhere. It is original and not being submitted to any other journal.

## AUTHOR CONTRIBUTIONS

Tiangui Yang, Peng Fu, Jie Chen and Xiaoxia Liu wrote the manuscript and researched the data. Changlu Xu, and Tiesheng Niu contributed to discussion. Tiangui Yang, Xi Fu and Tiesheng Niu designed the study and reviewed the data and revised the manuscript. All authors read and approved the final manuscript.

## ETHICS STATEMENT

This study was approved by the Ethics Committee of Shengjing Hospital. Written informed consents was obtained from all enrolled MI inpatients.

## Supporting information


**Figure S1 Various sorts of fragmented QRS.** Characteristics of fQRS: ① QRS waves are three‐phase or polyphase waves, some of which are typical of RSR type where polyphase waves are often formed by multiple setbacks or notches of R waves or S waves; ② with or without Q waves, they can form QR or QR type; ③ most of QRS wave time limit <120 ms; ④ except complete or incomplete bundle branch block and indoor conduction block; ⑤ three‐phase or multi‐phase QRS fragmentation often occurs in two or more leads corresponding to the coronary blood supply area; ⑥ different leads of the same ECG in the same patient may show different forms of QRS fragmentation.

**Figure S2 The coronary angiography images before and after PCI of two patients who had fQRS on ECG.** These two patients had typical fQRS on ECG (A1, B1, red arrow). The angiogram indicates the corresponding CTO coronary before PCI (A2, CTO of RCA; B2, CTO of LAD, blue arrow), the Rentrop grade of CCC in both cases were grade 3 (A3, B3, blue arrow), and the blood forward flow was TIMI 3 after PCI (A4, B4, blue arrow). The cardiac function of both cases significantly improved after PCI (A5, B5). PCI: percutaneous coronary intervention; CTO: chronic total occlusion; CCC: coronary collateral circulation; LAD: left anterior descending; RCA: right coronary artery; 6MWT:6‐minute walking test; BNP: brain natriuretic peptide; LVEF: left ventricular ejection fraction; LVEDVI: left ventricular end‐diastolic volume index.

## Data Availability

All data generated or analyzed during this study are included in this article.
