# Supplementary figures and images for: The value of fragmented QRS in predicting the prognosis of chronic total occlusion patients with myocardial infarction history undergoing percutaneous coronary intervention: A 24‐months follow‐up study
Source: Clin Cardiol. 2021 Feb 16;44(4):537–46. doi: 10.1002/clc.23573 (PMC8027581; doi:10.1002/clc.23573)

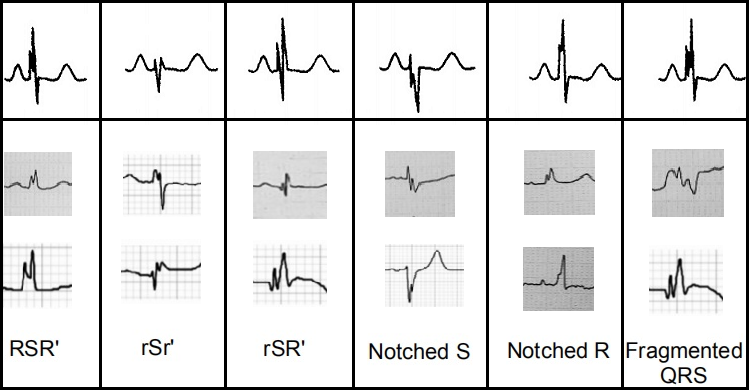

Supplement: Supplementary file 1 — Figure S1 Various sorts of fragmented QRS. Characteristics of fQRS: ① QRS waves are three‐phase or polyphase waves, some of which are typical of RSR type where polyphase waves are often formed by multiple setbacks or notches of R waves or S waves; ② with or without Q waves, they can form QR or QR type; ③ most of QRS wave time limit <120 ms; ④ except complete or incomplete bundle branch block and indoor conduction block; ⑤ three‐phase or multi‐phase QRS fragmentation often occurs in two or more leads corresponding to the coronary blood supply area; ⑥ different leads of the same ECG in the same patient may show different forms of QRS fragmentation. [file CLC-44-537-s001.tif]

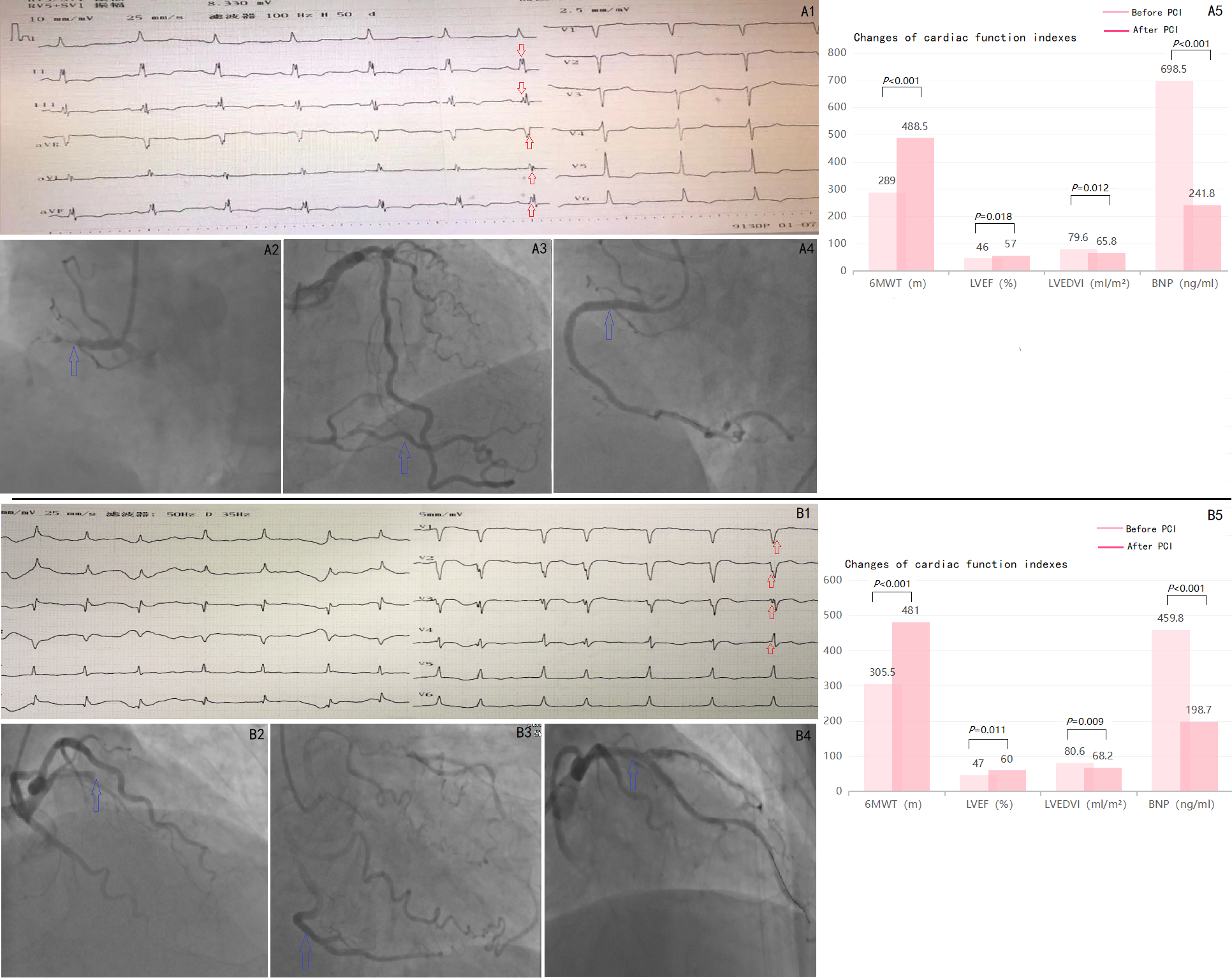

Supplement: Supplementary file 2 — Figure S2 The coronary angiography images before and after PCI of two patients who had fQRS on ECG. These two patients had typical fQRS on ECG (A1, B1, red arrow). The angiogram indicates the corresponding CTO coronary before PCI (A2, CTO of RCA; B2, CTO of LAD, blue arrow), the Rentrop grade of CCC in both cases were grade 3 (A3, B3, blue arrow), and the blood forward flow was TIMI 3 after PCI (A4, B4, blue arrow). The cardiac function of both cases significantly improved after PCI (A5, B5). PCI: percutaneous coronary intervention; CTO: chronic total occlusion; CCC: coronary collateral circulation; LAD: left anterior descending; RCA: right coronary artery; 6MWT:6‐minute walking test; BNP: brain natriuretic peptide; LVEF: left ventricular ejection fraction; LVEDVI: left ventricular end‐diastolic volume index. [file CLC-44-537-s002.tif]
